# Draft Genome Sequence of a Noncognate Bacterium, *Achromobacter* sp. Strain Bel, Associated with a Rhabditid Entomopathogenic Nematode

**DOI:** 10.1128/MRA.01247-20

**Published:** 2020-11-25

**Authors:** Don Mangowa, Mahloro H. Serepa-Dlamini

**Affiliations:** a Department of Biotechnology and Food Technology, University of Johannesburg, Johannesburg, South Africa; University of Maryland School of Medicine

## Abstract

*Achromobacter* sp. strain Bel is a noncognate bacterium associated with a rhabditid entomopathogenic nematode of the *Oscheius* genus. Its draft genome sequence is 6.62 Mb with a G+C content of 65.4%. The genome highlights important genes which could play a role in entomopathogenicity.

## ANNOUNCEMENT

Entomopathogenic nematodes (EPNs) are obligate insect parasites (1), which are associated with symbiotic bacteria which assist in killing the insect larvae (2). Entomopathogenic nematodes of the genera *Steinernema*, *Heterorhabditis*, and *Oscheius* are symbiotically associated with cognate symbiotic *Enterobacteriaceae* bacteria of the *Xenorhabdus*, *Photorhabdus*, and *Serratia* genera, respectively (3). Other noncognate bacterial species from genera such as *Pseudomonas*, Acinetobacter, *Staphylococcus*, *Stenotrophomonas*, *Ochrobactrum*, and *Achromobacter* have been isolated from EPNs (4–7). Although the function of noncognate bacteria is not fully delineated, some noncognate species have been reported to contribute toward the entomopathogenicity of EPNs (7). Thus, the genome sequences of noncognate bacteria will contribute toward (i) understanding their role toward EPN pathogenicity, (ii) knowing the impact they have on nematode fitness and reproduction, and (iii) knowing how their genomic profiles differ from cognate symbiotic bacteria with regard to infectivity against insects.

*Achromobacter* sp. strain Bel was isolated from infective juveniles (IJs) of an *Oscheius* EPN isolated from Bela-Bela, Limpopo Province, South Africa. In brief, collected soil samples were baited with Galleria mellonella larvae, and IJs were collected from white traps. The IJs were surface sterilized in 3% NaClO for 5 min and crushed with sterile pestle and mortar. The homogenate was streaked onto nutrient triphenyl-tetrazolium bromothymol blue agar (NBTA) plates and incubated at 28°C for 48 h. Bacteria were routinely grown in nutrient broth at 28°C; long-term preservation was in 30% glycerol (vol/vol), and samples were stored at −80°C.

Genomic DNA was extracted from solid bacterial colonies on the NBTA plates following the protocol of the NucleoSpin microbial DNA extraction kit (Macherey-Nagel, Germany). The genomic DNA was sent for sequencing at a commercial service provider, the Agricultural Research Council (ARC), Onderstepoort, Pretoria, South Africa. The genome was sequenced on the HiSeq 2500 Illumina platform; paired-end libraries (2 × 150 bp) were generated using the Nextera DNA sample preparation kit (Illumina, USA).

The raw genome sequences were subjected to quality control using FastQC v 0.69 (8) on a Web-based platform, Galaxy, which is available online at https://usegalaxy.org (9). The genome was *de novo* assembled using Unicycler v 0.4.1.1 (10) and assessed using QUAST v 4.6.3 (11). Default parameters were used, except where otherwise stated. The draft genome was annotated using the Prokaryotic Genome Annotation Pipeline (PGAP) available at NCBI (12).

The sequencing platform produced 3,140,073 sequence reads with 142× coverage. The *Achromobacter* sp. strain Bel draft genome size is 6,624,814 bp which yielded 121 contigs, with an *N*_50_ value of 100,154 bp and a G+C content of 65.4%. The total number of genes predicted were 6,166, including 6,047 protein-coding genes, 55 pseudogenes, 63 RNAs, 55 tRNAs, 4 noncoding RNAs (ncRNAs), and 3 rRNAs represented as 5S, 16S, and 23S. A number of genes that code for virulence factors, toxins, and enzymes, such as lipases and proteases which play a role in entomopathogenicity, were identified from the strain Bel genome; similar genes have previously been identified in EPN cognate symbiotic bacteria (13–15).

### Data availability.

This whole-genome shotgun project has been deposited at DDBJ/ENA/GenBank under the accession number JABBJN000000000, BioProject accession number PRJNA625632, and BioSample SAMN14604087. The raw sequence reads are available at PRJNA625632. The version described in this paper is the first version, JABBJN010000000.
